# Data on the angular characteristics of the spiral structure of the narwhal tusk

**DOI:** 10.1016/j.dib.2018.09.029

**Published:** 2018-09-15

**Authors:** Andrey Bondoevich Kiladze, Olga Fedorovna Chernova

**Affiliations:** A.N. Severtsov Institute of Ecology and Evolution, Russian Academy of Sciences, Moscow, Russia

**Keywords:** The Narwhal *Monodon monoceros*, The narwhal tusk, Cementum layer, Spiral, Tangent of the angle of inclination, Angle of inclination

## Abstract

We studied morphometric features of the spiral structure of the narwhal tusk and show that angular parameters of the arcs of the cementum layer determine the pattern of the spiral structure of the tusk. Given the straight shape of the tusk and the inclined pattern of the arcs of the cementum layer, the angular characteristics of the spiral were determined through the ratio of the legs of a right triangle, which was a basis for calculating the tangent of the angle of inclination. We found the angle of inclination through the arctangent. The tangent of the angle of inclination of the arcs of the spiral of the narwhal tusk is 2.39 ± 0.07, while the angle of inclination is 66.88 ± 0.61°. The indicators reflect the spiral morphology of the narwhal tusk. Researchers can use the data obtained in morphology practices, as well as in modeling the natural spiral structure in biomimetics.

**Specifications table**

**Table:** 

Subject area	Cetology
More specific subject area	Morphology of the Narwhal *Monodon monoceros*
Type of data	Text, figure, table
How data was acquired	The data were obtained by geometrical and statistical methods
Data format	Visual and statistical analyzed data
Experimental factors	The data were obtained from the museum collection of narwhal tusks
Experimental features	The angular parameters were estimated by proportional ratios determined from the photographs
Data source location	The Zoological Museum of the Zoological Institute, the Russian Academy of Sciences, Saint Petersburg, Russia (ZIN)
Data accessibility	Data is with this article
Related research article	*—*

**Value of the data**

• Zoologists can use the data obtained in morphological studies of the spiral structure of the narwhal tusk.
• Biophysicists and engineers can used the data obtained for modeling the natural texture of the narwhal tusk.
• The data obtained exemplifies shape formation in nature that is why practical significance of the results is associated with the use of data in biomimetics.

## 1 Data

**Fig. 1:**
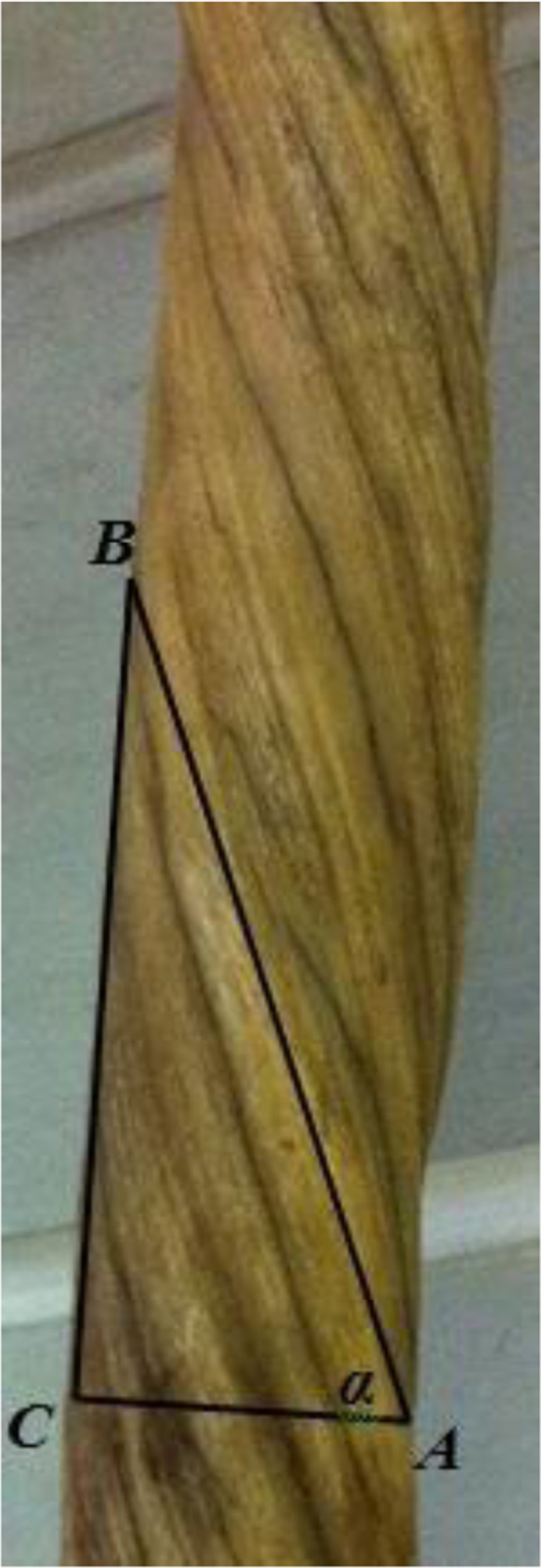
Determination of the angular parameters of the spiral structure of a tusk of the Narwhal *Monodon monoceros*: *AB* — the spiral arc; *AC* — the diameter of the tusk; *BC* — arc length of a spiral; *α* — the angle of inclination. ZIN. Photograph by A.B. Kiladze.

The typical narwhal tusk is an elongated spindle-shaped straight tooth, whose structure is distinguished by a clear spiral system [1], [2], [3], [4], [5] of deposition of the surface cementum layer (Fig. 1).

The orientation of the twisting is oblique, which indicates the elongation of the spiral along the long axis of the tusk. To determine the angle of inclination of the spiral arc of the tusk, we estimated the inclination angle tangent equal to the ratio of the arc length of a spiral of the tusk (Fig. 1, *BC*) to the diameter of the tusk (Fig. 1, *AC*): tan α = *BC* / *AC*.

To find the angle of inclination, the arctangent was determined. The data in Table 1 indicate a more than twofold increase in the arc length of a spiral above the diameter of the tusk. This relationship forms the corresponding angle of inclination of the spiral arc, which determines the longitudinal elongation of the spiral structure of the tusk.

**Table 1:** Angular parameters (*n* = 30) of the arcs of a spiral of a tusk of the Narwhal *Monodon monoceros*.

Angular parameters of the spiral arc	Statistical parameters		
	*M* ± *m*^a^	Minimum	Maximum
Tangent of angle of inclination (tan *α*)	2.39 ± 0.07	1.65	3.22
Angle of inclination (*α*), °	66.88 ± 0.61	58.78	72.75

a Note: *n* is the number of measurements; *M* ± *m* is the arithmetic mean with the error of the arithmetic mean.

## 2 Experimental design, materials, and methods

Six tusks of the Narwhal *Monodon monoceros*, housed in the Zoological Museum of the Zoological Institute of the Russian Academy of Sciences (St. Petersburg), were investigated. The tusks were photographed. Five measurements were made from the base to the middle of each tusk. The total number of measurements is 30 (*n* = 30). The angular parameters were calculated not by absolute values, but by proportional ratios, which were determined from the photographs. Taking into account the general straightness of the tusk and the sloping character of the outer cementum layer, the angular parameters were calculated by determining the ratio of the length of the legs of the rectangular triangle, i.e. by means of a trigonometric function. For this purpose, we determined the tangent of the angle of inclination, which has a dimensionless character. Arctangent was estimated with the scientific calculator "CITIZEN SRP-285II" (Citizen Systems Japan Co., Ltd, Made in China) to determine the angle of inclination in degrees (°).

For the control purposes, the angle of inclination of the arcs of the spiral was determined with the help of a protractor (the method of direct angular detection). The angles determined by these two methods practically coincided.

The obtained data was processed by the methods of descriptive statistics. The data files were statistically processed on the computer program "STATISTICA 10" (StatSoft, USA).
